# Investigating the Effect of Various Sous-Vide Cooking Conditions on Protein Structure and Texture Characteristics of Tilapia Fillet Using Synchrotron Radiation-Based FTIR

**DOI:** 10.3390/foods12030568

**Published:** 2023-01-28

**Authors:** Jaksuma Pongsetkul, Supatcharee Siriwong, Kanjana Thumanu, Surintorn Boonanuntanasarn, Jirawat Yongsawatdigul

**Affiliations:** 1 School of Animal Technology and Innovation, Institute of Agricultural Technology, Suranaree University of Technology, Nakhon Ratchasima 30000, Thailand; 2 Synchrotron Light Research Institute (Public Organization), Nakhon Ratchasima 30000, Thailand; 3 School of Food Technology, Institute of Agricultural Technology, Suranaree University of Technology, Nakhon Ratchasima 30000, Thailand

**Keywords:** sous vide, texture, synchrotron FTIR, Nile tilapia, principal component analysis

## Abstract

The effects of various sous-vide (SV) cooking conditions (50-60℃, 30-60 min) on physicochemical properties related to the texture characteristics, protein structure/degradation, and sensory acceptability of tilapia fillet (*Oreochromis niloticus*) were investigated. With an increasing temperature and processing time of SV cooking, protein degradation (of both myofibrils and connective tissue) was more pronounced, as evaluated by the decrease in water- and salt-soluble proteins, total collagen, as well as the changes in the ratio of secondary protein structures (α-helix, β-sheet, β-turn, etc.), which were determined by synchrotron-FTIR (SR-FTIR). These degradations were associated with the improvement of meat tenderness, as estimated by shear force and texture profile analyzer (TPA) results. Among all SV conditions, using 60 ℃ for 45 min seems to be the optimal condition for tilapia meat, since it delivered the best results for texture characteristics and acceptability (*p* < 0.05). Moreover, principal component analysis (PCA) results clearly demonstrated that the highest texture-liking score of this condition was well associated with the intensity of β-sheets, which seem to be the crucial component that affected the texture of SV-cooked tilapia more so than other parameters. The findings demonstrated the potential of SR-FTIR to decipher the biomolecular structure, particularly the secondary protein structure, of SV-cooked tilapia. This technique provided essential information for a better understanding of the changes in biomolecules related to the textural characteristics of this product.

## 1. Introduction

Texture, both tenderness and juiciness, is an important trait in meat that is associated with high quality, influencing the overall likability of meat products. Generally, meat tenderness is influenced by the amount and solubility of connective tissue, the presence of intramuscular fat (marbling), as well as postmortem proteolysis of myofibrillar and myofibrillar-associated proteins [1]. Meat juiciness, a uniquely subjective property of meat, is the impression of wetness during the first few chews, produced by the rapid release of meat fluids [2]. It is in part determined by water-holding capacity (WHC). The effect of heat applied to muscle to prepare meat for consumption is of importance for these characteristics. During heating, muscle proteins denature, solubilize, and form gels, depending on the heating conditions applied, with effects on both meat tenderness and WHC, determining the sensory acceptability of the cooked meat products [3].

Sous-vide (SV) technology is one of the modern cooking techniques that assures a reliable and constant quality that is in full compliance with the market demands for rigorous sanitary standards, thus increasing the popularity over the world’s kitchen [4]. It refers to the method of cooking vacuum-packed food under precise temperature and time control [5]. Compared with conventional cooking methods, SV typically involves cooking at relatively low temperatures (50–80 °C) for a longer period, resulting in preserving the food’s nutritional value and sensory characteristics, particularly texture [6]. Over the last decade, using the SV technique to improve the meat quality of cooked fish, i.e., salmon (*Salmon salar*) [7], tuna (*Thunnus maccoyii*) [8], trout (*Oncorhynchus mykiss*) [9], emperor bream (*Lethrinus lethrinus*) [10], tilapia (*Oreochromis niloticus*) [11], etc., has been achieved. However, these studies were mostly focused only on quality attributes and microbiological safety. Until now, there has been little information reported on the in-depth association between sous-vide (SV) conditions (temperature and time), physicochemical properties, biomolecular structure, and sensory acceptability. Further, studies devoted to assessing the SV cooking technique with Nile tilapia (*O. niloticus*), one of the most popular fish species consumed in the world, particularly in Asia, are still scarce.

Understanding the structural changes of the whole protein may insightfully demonstrate the understanding of its effects on meat texture and consumer acceptability as affected by heat treatment level. In this study, synchrotron radiation-based Fourier-transform infrared (SR-FTIR) microspectroscopy was used as a tool to investigate this knowledge. SR-FTIR can precisely explore the molecular chemistry within the microstructures of biological samples with high signal-to-noise ratios at ultra-spatial resolutions because of its high light brightness (which is usually 100–1000 times brighter than a conventional globar source and has a small effective source size). This technique can provide quantitative information on the secondary structure of proteins, which leads to an understanding of the components that make up a whole protein [12]. Therefore, the objective of this study was to investigate the relationship between the mechanisms of muscle protein degradation, some crucial physiological properties underlying meat texture, and the consumer acceptability of various SV-cooked tilapia fillets using SR-FTIR in combination with principal component analysis (PCA).

## 2. Materials and Methods

### 2.1. Sample Preparation

Nile tilapia (*O. niloticus*) with an average weight of 656.25 ± 82.26 g were brought on ice to the laboratory from 3 shops in the fish market (3 shops represented 3 lots of samples) (150 fish/lot). Upon arrival, fish were washed with chilled water and dressed into fillets of 1.0-1.5 cm thickness (each weighing about 150 g) (300 fillets/lot). The fish fillets were then individually vacuum-sealed using a vacuum-packing machine (FVC-II, Furukawa MFG Co., Ltd., Japan) with a 99.6% extent of vacuuming. Samples were randomly divided into 6 groups (approximately 40 fillets/group) and cooked using the sous-vide (SV) technique by immersing them in a continuously thermocontrolled water bath under various temperatures and times, including (1) 50 °C for 30, 45, and 60 min (5–30, 5–45, and 5–60) and (2) 60 °C for 30, 45, and 60 min (6–30, 6–45, and 6–60). During SV cooking, a temperature data logger series II (ThermaData-K, Chandler, AZ, USA) with an embedded thermocouple probe was inserted into vacuum pouches with the tilapia fillets to monitor the fluctuations in temperature. The final temperature of core fillets of 5–30, 5–45, 5–60, 6–30, 6–45, as well as 6–60 was 48, 50, 50, 56, 60, and 60 °C, respectively (temperature profiles of heating medium and core tilapia fillets during SV cooking are reported as Supplementary Figure S1). After SV cooking, samples were then rapidly cooled down with tap water and subjected to the following experiments. For each parameter, the experiment was performed with 3 replicates from 3 fillets of each lot and reported as the mean ± S.D (*n* = 9).

### 2.2. Physicochemical Properties of SV-Cooked Tilapia Fillet

#### 2.2.1. Cooking Loss/Water-Holding Capacity (WHC)

WHC was determined using a low-speed centrifugation (1000× *g* for 5 min, 4 °C) method as described by Digre et al. [13] and expressed as the percentage of water retained in the mince after centrifugation. Cooking loss was evaluated as the reduction in weight during SV cooking. The treated sample was wiped gently with tissue paper to remove excess surface moisture, before performing the measurements as a percentage of the initial weight (*w*/*w*, wet basis) [13].

#### 2.2.2. Shear Force and Texture Profile Analysis (TPA)

SV-cooked tilapia fillets were cut into small pieces (1 × 2 × 1 cm^3^, width × length × thickness) to analyze their shear force and TPA. For shear force, samples were measured using a Warner–Bratzler blade equipped with a texture analyzer (TA.XT.Plus, Stable Micro Systems, Surrey, UK) with a speed test of 4 mm/s and a 50 kg load cell [14]. TPA was measured on a texture analyzer equipped with a 1 kg load cell. A flattened cylinder of 12 mm in diameter was compressed into the fish fillet at a constant speed of 1 mm/s until it reached 60% of its original height, carefully avoiding myocommata. The holding time between the compressions was 5 s. Bluechill 2 software was used to collect and process the data. The results were expressed as hardness (N), chewiness (N), springiness (mm), and cohesiveness (ratio) [15].

### 2.3. Protein Structure/Degradation of Various SV-Cooked Tilapia Fillets

#### 2.3.1. Water- and Salt-Soluble Protein Content

The content of sarcoplasmic (water-soluble) and myofibrillar (salt-soluble) proteins was determined as per the method of Hultmann and Rustad [15] with slight modification. A ground sample (4 g) was mixed with 80 mL of 0.05 M phosphate buffer (pH 7.0) and homogenized for 1 min using an IKA Labortechnik homogenizer (Selangor, Malaysia) at a speed of 9500× *g*. The homogenate was then centrifuged at 10,000× *g* for 20 min (4 °C) using a refrigerated centrifuge (Beckman Coulter, Avanti J-E Centrifuge, Palo Alto, CA, USA). The supernatant was collected, and the volume was adjusted to 100 mL with phosphate buffer (water-soluble fraction). The remaining precipitate was further homogenized for 10 s in 0.05 M phosphate buffer containing 0.6M KCl and 0.5% tritonX-405 (pH 7.0), followed by centrifugation as described above. The supernatant was collected and adjusted to 100 mL with KCl-phosphate buffer (salt-soluble fraction). Protein contents in the water- and salt-soluble extracts were determined using the BioRad protein assay with gamma globulin as a standard and expressed as a percentage (%) of the wet weight sample.

#### 2.3.2. Total and Insoluble Collagen Content

Collagen content was determined according to the method of Kolar [16] with slight modification. For total collagen, a sample (2 g) was hydrolyzed with 7N H_2_SO_4_ at 105 °C for 16 h. The hydrolysate was neutralized with 3.5M H_2_SO_4_, filtered, and then added with chloramine-T solution and Ehrlich’s reagent to react. The absorbance was measured at 550 nm using hydroxyproline (Sigma-Aldrich Co., St. Louis, MO, USA) as a standard. Total collagen content was calculated using a conversion factor of 7.25 and expressed as mg/g sample.

For insoluble collagen content, a fish sample (2) was first added to 20 mL of Ringer’s solution (32.8 mM NaCl, 1.5 mM KCl, and 0.5 mM CaCl_2_), homogenized, and then heated at 77 °C for 70 min. The homogenate was centrifuged, and residual fractions were further hydrolyzed in 7N H_2_SO_4_ at 105 °C for 16 h. The hydroxyproline content of the hydrolysate was determined after neutralization [16].

#### 2.3.3. Scanning Electron Microscopy (SEM)

The microstructure of fish samples was determined as per the method of Wan et al. [17] with slight modification. Fish samples were cut into cubes (1 × 2 × 1 cm^3^, width × length × thickness) and fixed with 2.5% glutaraldehyde solution (*v*/*v*) in 0.2 M phosphate buffer (pH 7.2) at 4 °C for 24 h, followed by rinsing with distilled water. Fixed samples were dehydrated in ethanol with serial concentrations of 25, 50, 70, 80, 90, and 100% for 15 min each time. Samples were critical-point-dried using CO_2_ as a transition fluid. The prepared samples were mounted on a bronze stub and sputter-coated with 10 nm of gold. Samples were visualized using SEM (S-3400N, Hitachi, Arisona, USA) with 3.0 kV operating voltage and 4.2 work distance at 5000 magnifications.

#### 2.3.4. Synchrotron-Based Fourier-Transform Infrared Spectroscopy (SR-FTIR)

SV-cooked samples were cut into pieces that were 0.5 × 0.5 cm^2^ and embedded in a foil block containing an optimal cutting temperature (OCT) compound. Then, samples were frozen immediately in liquid nitrogen. The OCT compound was used to avoid causing freezing damage to the samples. The samples were cross-sectioned to 5 µm via a MicromTM HM 525 Cryostat at −22 °C, then put on a transparent BaF_2_ IR window and stored in a desiccator before analysis. Each SV cooking condition was replicated six times.

The biomolecule spectra of various SV-cooked samples were determined using FTIR spectroscopy (Vertex 70) coupled with an IR microscope (Hyperion 2000, Bruker Optics, Ettlingen, Germany. OPUS software (version 7.8, Bruker Optics Ltd., Ettlingen, Germany) was used to acquire the spectra and to control the instrument. The spectral data were collected from 4000 to 400 cm^–1^ with a spectral resolution of 4 cm–1 with 64 scans co-added. In each sample, 30 spectra were collected for each replication to obtain a total of 180 spectra. Spectra were processed using the Unscrambler X software (version 10.5, Camo Analytics, Oslo, Norway) by considering the second derivative with third-order polynomial using the Savitzky–Golay algorithm with 13 points of smoothing, allowing for the minimization of the effects of variable baselines. The spectra were vector normalized to cover the region of 3000–2800 cm^–1^ and 1800–900 cm^–1^ to focus on the changes in these regions, which can help resolve nearby peaks and sharpen spectral features. The variability within the spectral data was analyzed using principal component analysis (PCA) and was explained into the first few principal components (PCs).

The intensity of interested bands of biomolecules, including 3000–2800 cm^–1^ (C-H stretching from lipid), 1740 cm^–1^ (C=O ester from lipid), 1700–1600 cm^–1^ (amide I), 1600–1500 cm^–1^ (amide II), 1338 cm^–1^ (amide III), as well as 1250–900 cm^–1^ (carbohydrate and glycogen), was determined and expressed as a percentage of integral area. In addition, protein secondary structures were determined as percentages of α-helix, β-sheet, β-turn, and antiparallel conformations based on curve fitting in the 1700–1600 cm^–1^ (amide I) range using the appropriate Gaussian and Lorentzian functions in OPUS 7.8 software (Bruker Optics Ltd., Ettlingen, Germany).

### 2.4. Texture-Liking Score

The texture-liking score of various SV-cooked tilapia was conducted using a 9-point hedonic scale to assess consumer acceptability. Seventy-five untrained panelists (>18 years old) who enjoy consuming tilapia were asked to assess the liking scores of texture characteristics (1 = extremely dislike, 9 = extremely like). The samples were mildly heated for 45 s using a microwave oven MW 206 of 200 watts and a frequency of 2450 MHz (EMM20K18GW, Electrolux, Thailand) for equally hot serving of the samples. Then, each fillet was divided into 4 pieces, and each piece was labeled with a 3-digit random code and randomly served to the panelists to assess the score. The evaluation was conducted in individual sensory evaluation booths under fluorescent white light. Panelists were instructed to evaluate the samples and take a sip of water after testing each sample.

### 2.5. Statistical Analysis

The results were expressed in means ± standard deviation (SD). The data obtained were analyzed by one-way analysis of variance using the SPSS package version 20.0 (SPSS for Windows, SPSS Inc., Chicago, IL, USA). All mean separations were carried out by Duncan’s multiple range test (DMRT) using a significance level of 5% (*p* < 0.05). For multivariate data analysis, a matrix of data on physicochemical properties, sensory scores, as well as secondary protein structures from SR-FTIR was created. The clustering of the variables was then analyzed using principal component analysis (PCA). The relationships between variables were identified simultaneously using bi-plots obtained by calculations from a two-dimensional scatter plot of PCA with the dominant spectral band of the different variables.

## 3. Results and Discussion

### 3.1. Physicochemical Properties of SV-Cooked Tilapia Fillet

#### 3.1.1. WHC/Cooking Loss

**Figure 1:**
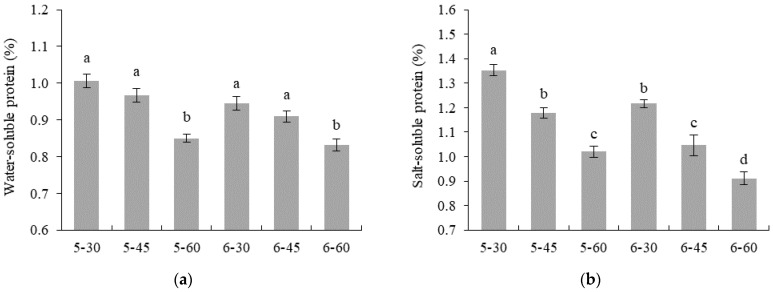

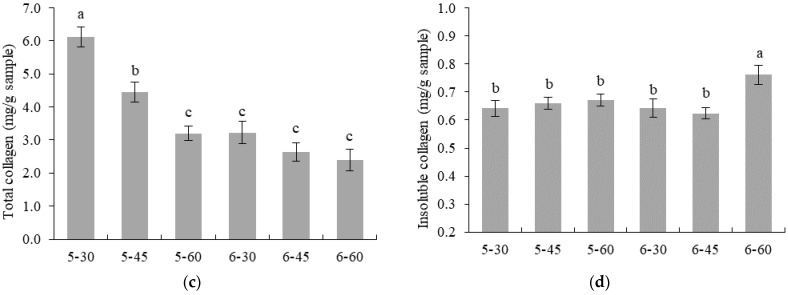
Water-soluble protein (**a**), salt-soluble protein (**b**), total collagen (**c**), and insoluble collagen (**d**) of tilapia fillets processed under various SV conditions. Different lowercase letters indicate significant differences (*p* < 0.05).

The WHC of tilapia fillets decreased from 93.63% (Supplementary Table S1) to 70.32–82.75% after SV cooking was applied, as shown in Table 1. Among all samples, the lowest WHC was found in 6–60, and the highest WHC was found in 5–30 (*p* < 0.05). It was clearly observed that a higher temperature or longer treatment time of SV cooking resulted in more liquid loss from the meat structure. The results agreed well with the cooking loss. It was found that the highest cooking loss was obtained (9.31%) when the fish was processed with the most severe SV conditions (6–60) (*p* < 0.05). Baldwin [4] has stated that SV-induced denaturation of the muscle protein apparently caused shrinkage of the muscle fibers that resulted in extruding the water held between the myosin and actin filaments of the myofibrils. This corresponds well with the greater degradation of the salt-soluble protein seen in samples treated with severe SV conditions, particularly 6–60 (Figure 1B). Water loss has considerable economic implications for cooked fish, since it is sold by weight, and retention of water is equally important for the optimal texture of muscle because of its substantial impact on meat tenderness and juiciness [6]. Moreover, water, which gets lost in processing, can take away many water-soluble compounds, such as salts, proteins, polyphosphates, and many aromatic compounds, affecting the product’s flavor [18].

#### 3.1.2. Shear Force and Texture Profile Analysis (TPA)

The shear force of the SV-cooked tilapia ranged from 7.98–9.25 N (Table 1). Samples treated with 60 °C had a lower shear force than samples treated with 50 °C (*p* < 0.05). For 50 °C SV-cooked fish, a decrease in shear force was observed when increasing the treatment time (*p* < 0.05). However, there was no significant difference in shear force among samples treated at 60 °C (*p* > 0.05). Generally, the solubilization of connective tissue in the temperature range of 50–70 °C leads to flesh tenderization [4]. Vaudagna et al. [19] revealed a positive correlation with beef loin tenderness when processing temperatures of SV cooking were increased from 50 °C to 65 °C. In this experiment, the results corresponded well with the changes in collagen content (Figure 1C), which showed that higher temperatures of SV treatment (60 °C) significantly affected the higher protein degradation (particularly collagen), resulting in more tenderness of the fish flesh. This may be associated with the improvement of consumer satisfaction of texture characteristics, as indicated by the higher texture-liking score of tilapia cooked at 60 °C (Table 1).

TPA results are presented in Table 1. As the temperature and processing time of SV increased, most texture parameters, including the hardness, chewiness, and springiness of SV-cooked tilapia, decreased (*p* < 0.05). This indicated a gradual softening of muscles when applied to more severe SV conditions. The hardness of fresh tilapia was 1368.01 ± 103.25 N (Supplementary Table S1). This value then decreased to 372.79–632.81 N after application with SV cooking. It was observed that samples treated at 60 °C had significantly lower hardness than samples treated at 50 °C (*p* < 0.05), corresponding well with the lower shear force. This indicated that cooking fish at 60 °C can improve meat tenderness better than 50 °C, particularly when treated for up to 1 h. Normally, the first perception of texture related to the first bite is usually associated with hardness [20]. Then, during chewing, the structure of the meat matrix continues to change its physical characteristics, and the hardness decreases. In this later period, the tenderness/chewiness, which would be experienced with the breakdown of muscle fiber and fat and the release of water contents, plays a role in texture perception. Chen et al. [21] suggested that tenderness/chewiness is another time-dependent evolution, and WHC is positively correlated with succulent attributes during evaluation. In this study, the results of chewiness were similar to hardness. It was found that lower chewiness was observed when the fish was applied to more severe SV conditions. In fact, chewiness, which can indicate the number of chews required before swallowing, is the result of the combined effects of hardness, cohesiveness, and springiness characteristics. A positive correlation between chewiness and hardness in cooked meat products was pronounced in the study of Pematilleke et al. [22]. For cooked meat, hardness and chewiness have greater variation compared to other characteristics, i.e., springiness, cohesiveness, etc., thus providing greater impact on the texture changes in the final product [22]. For other characteristics, it was found that the springiness of SV-cooked tilapia showed a similar trend as hardness and chewiness, in which a decrease in values was obtained when temperature and time for SV were increased (*p* < 0.05). Meanwhile, there was no difference in cohesiveness among all 6 samples (*p* > 0.05). Overall, from our results, it could be claimed that using 60 °C for SV cooking, particularly when extending the processing time, can improve the initial bite tenderness (as indicated by lower shear force and hardness) and may further allow the meat to require less mastication effort (as indicated by lower chewiness). However, unfortunately, because the amount of mastication was not a focus of this study, our results were speculative at best and in relation to the hardness or initial bite only. The results agreed well with the study of Yao [23], who found that the hardness of fish meat was gradually reduced when it was exposed to a prolonged SV cooking time at 60 °C up to 4 h. It was also found that SV cooking can effectively soften fish meat, compared with traditional cooking.

### 3.2. Protein Structure/Degradation of Various SV-Cooked Tilapia Fillets

#### 3.2.1. Water- and Salt-Soluble Protein Content

Fresh tilapia meat contained water-soluble (or sarcoplasmic) and salt-soluble (or myofibrillar) proteins of 5.23 and 10.06%, respectively (Supplementary Table S1). Both water- and salt-soluble proteins decreased to 0.83–0.98 and 0.91–1.35%, respectively, after application with SV cooking, as shown in Figure 1a,b. This reduction, accounting for more than 80%, indicated heat denaturation and the aggregation of these proteins caused by all SV conditions. It was found that both water- and salt-soluble proteins decreased when using higher temperatures for SV cooking and when processed for a longer time, with the lowest content obtained in 6–60 (*p* < 0.05). The results agreed with those of Cropotova et al. [24], who reported that a decrease in water- and salt-soluble proteins of SV-cooked mackerel (*Scomber scombrus*), processed at 60–90 °C for 10–20 min, was observed as the temperature and treatment time increased. Naveena et al. [25] also stated that SV-cooked chicken showed a marked increase in the denaturation of proteins when cooked at 100 °C for 60 min and 120 min, compared with 30 min. Normally, myofibrillar proteins are the major protein, and their network is generally responsible for holding water inside the fish muscle [15]. Thus, the decrease in solubility of salt-soluble proteins may contribute to the decrease in WHC or increase in cooking loss due to thermal denaturation and the aggregation of myofibrils. In this study, therefore, the lowest salt-soluble protein of 6–60 coincided well with the lowest WHC and the highest cooking loss (Table 1).

#### 3.2.2. Total and Insoluble Collagen Content

In general, fish muscles are mainly composed of myofibrils and surrounding intramuscular connective tissues, which have collagen as a major component [26]. An association between the amount and solubility of collagen and the meat texture, particularly of cooked meat, has been noted [27]. Similar to the changes in water- and salt-soluble proteins, the total collagen in tilapia sharply decreased from 12.19 mg/g sample (Supplementary Table S1) of raw meat to 2.39–6.13 mg/g sample after application with various SV cooking conditions (Figure 1c). When SV cooking at 50 °C was applied, the total collagen content significantly decreased as processing time increased (*p* < 0.05). Meanwhile, there was no significant difference in total collagen content between samples treated at 60 °C for 30–60 min (*p* > 0.05). This suggests that the temperature used for SV provides more impact on the decrease in the amount of total collagen, compared with the duration of SV cooking. Further, SV cooking at 60 °C, even for a short time, can intensively destroy collagen. Normally, an increase in the solubilization of collagen is obtained when mild heat (40–50 °C) is applied, particularly using the LTLT technique, with the effect of lowering the meat’s toughness [28]. Christensen et al. [29] reported that SV cooking had a positive correlation with solubilized collagen and tenderness. However, collagen shrinkage can take place when the meat is cooked at high a temperature, approximately 60–70 °C, which is associated with an increase in insoluble collagen and may accelerate the toughness of the meat [28]. In this study, the highest insoluble collagen was obtained in 6–60 (*p* < 0.05), accounting for 0.76 mg/g sample, and there was no difference among other samples (0.62–0.67) (*p* > 0.05) (Figure 1d). The lowest total collagen but highest insoluble collagen of 6-60 suggested more intensive collagen degradation, reflecting the loss of solubilized collagen caused by more intensive SV conditions. This was in accordance with the lowest shear force of 6–60 (Table 1). The decreased shear force due to an increase in insoluble collagen observed in the present study could be partly due to a decrease in collagen solubility expressed by a change in the ratio of heat-stable to heat-liable crosslinks in the collagen [30]. Overall, more severe SV conditions, both temperature levels and processing duration, result in more protein degradation, including myofibrillar, sarcoplasmic, as well as connective tissue. The different degrees of protein degradation caused by various SV conditions may further affect the difference in texture or other meat characteristics, which determines the consumer acceptability of the product, to some extent.

#### 3.2.3. Microstructure

**Figure 2:**
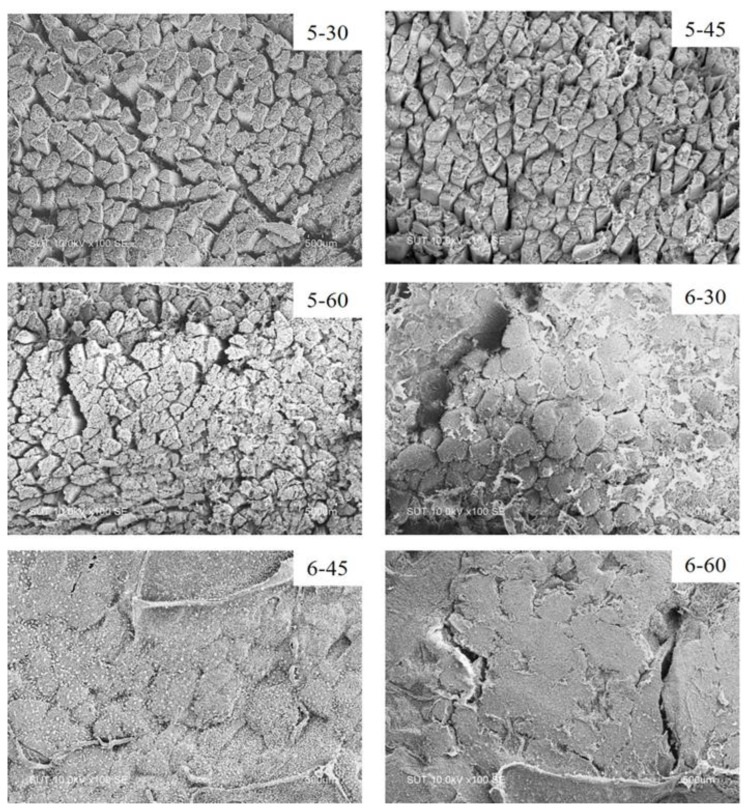
Scanning electron microscopy (SEM) images of tilapia fillet processed under various SV conditions (magnification: ×100).

The microstructures of SV-cooked tilapia processed by various conditions were visualized by SEM, as shown in Figure 2. Gaps between muscle fibers and bundles were visible in samples cooked at 50 ℃ (5–30, 5–45 and 5–60). Among all 50 ℃ SV-cooked tilapia, a denser structure was noticeable when the fish was processed for a longer time. When the proteins underwent thermal denaturation, the water was less imbibed or bound in their structure. The release of water from protein molecules might facilitate the muscle fiber to align closely, leading to a more compact structure [31]. It was found that samples processed at 60 °C for 30 min (6–30) had more compact fiber arrangement, as manifested by the lesser amount of gaping of the muscle fiber and bundle. The muscle fiber and bundles were torn and disappeared when processed for a longer time (6–45 and 6–60), indicating the destruction of connective tissue and sarcolemma membrane surrounding each fiber or bundle caused by more severe SV conditions. This correlated well with the lower total collagen, but higher insoluble collagen contents of 6–45 and 6–60 (Figure 1c,d). More pronounced protein degradation of these samples could be associated with more tenderness in texture, as indicated by a lower shear force (Table 1). Roldan et al. [32] reported that gaps between the muscle fibers were observed when processing lamb loin at 60 °C. These gaps became denser and more compact when the temperature of SV was increased to 70 and 80 °C.

#### 3.2.4. SR-FTIR Characteristics

**Figure 3:**
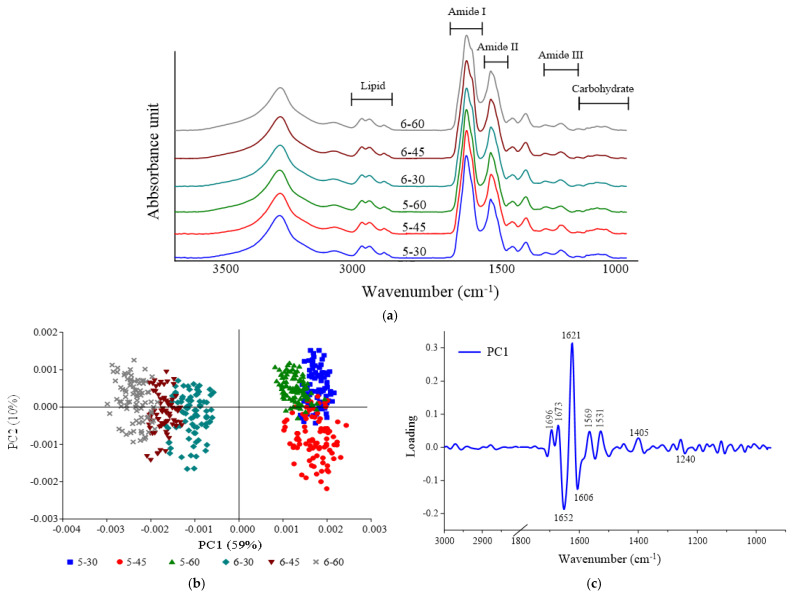
Average original spectra (**a**), PCA score plot (PC1 vs. PC2) (**b**), and correlation loading plot (PC1) (**c**) of some crucial spectra (3000–2800 cm^−1^ and 1800–900 cm^−1^) of SV-cooked tilapia fillets processed under various conditions.

Figure 3a shows the average FTIR spectra in the fingerprint region of wave numbers from 3000 to 900 cm^–1^ of cooked tilapia processed under various SV conditions. The average spectra from each sample revealed distinct differences in the peak heights and peak ratios of biomolecules, including lipids (3000–2800 cm^−1^), ester lipids (1740 cm^−1^), amide I (1700–1600 cm^−1^), amide II (1600–1500 cm^−1^), amide III (1338 cm^−1^), as well as carbohydrates/glycogen (1250–900 cm^−1^), and their integrated areas (%) are provided in Table 2. Six clustering of spectra were clearly visualized in a PCA score plot, which explained about 69% of the total variability of all data (Figure 3b). It was found that PC1, retaining about 59% of data variation, carried the major contribution of separating samples according to the level of temperature used for SV cooking. Samples treated at 50 °C for 30, 45, or 60 min were found on the right side of PC1, whereas samples treated at 60 °C were displayed on the left side of PC1 (the correlation loading plot of selected spectra, including 3000–2800 cm^−1^ and 1800-900 cm^−1^,mare given in Figure 3c), suggesting that changes in the biomolecules of SV-cooked fish were completely differentiated by the temperature used. In contrast, using different processing times (30–60 min) for SV cooking did not distinguish correlatively by those spectra. The spectra results highlighted that temperature had more impact on the quality of SV-cooked fish than processing time in this present study.

**Figure 4:**
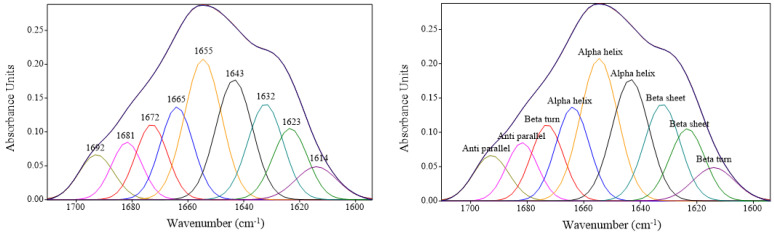
The curve fitting of amide I and secondary structure protein band assignment in tilapia fillets processed under various SV conditions.

Monitoring the changes in the structure of proteins, particularly their secondary structure, to assess the protein denaturation or protein–protein interactions via thermal processing is one of the most prevalent applications of SR-FTIR [33]. Table 2 shows the ratios of amide I and amide II of various SV-cooked tilapia fillets, which are the most conspicuous bands representing protein structures. The amide I band (1700–1600 cm^−1^) is extensively used for quantification of the secondary structure and for determining conformational changes in proteins and peptides, mostly arising from the C=O stretching vibrations. Meanwhile, the amide II band (1600–1500 cm^−1^), which originates from in-plane N−H bending and C−N stretching vibrations, exhibits less protein conformational sensitivity, but is still useful, especially for monitoring side chains [34]. It was noticed that the changes in the amide I and amide II bands fluctuated when increasing the temperature or processing time of SV cooking. Interesting data were achieved when the proportion of the secondary structure calculated from the amide I spectral profile after curve fitting (Figure 4) was calculated and expressed in Table 2. It was found that the intensity of α-helix bands of samples treated at 50 ℃ was significantly higher than samples treated at 60 °C (*p* < 0.05). In contrast, samples treated at 50 °C exhibited a lower intensity of β-sheet bands, compared with samples treated at 60 °C (*p* < 0.05). However, different processing times (30–60 min) did not affect either α-helix or β-sheet bands when the fish was processed at the same temperature (*p* > 0.05). Normally, a positive correlation between the α-helix band and tenderness is noticed, and the hydrophobic interactions and the β-sheet structure have been positively correlated with meat toughness, as reported by Beattie et al. [33]. From the FTIR results, it seems like meat toughness was ameliorated when treated at 60 °C. This is contrasted with the results of other physicochemical properties related to meat texture, i.e., shear force and TPA, which showed that samples treated at the higher temperature (60 °C) seem to be more tender, compared with 50 °C-cooked samples. This may explain that the higher intensity of β-sheets in samples treated at 60 °C resulted in more neat and compact proteins, thereby avoiding looser structures and not negatively impacting meat toughness. This explanation was supported by Nian et al. [35], who stated that the compactness of the protein secondary structure is generally governed by both α-helix and β-sheets, whereas more antiparallel and β-turns reflect a looser structure. In this study, there was no difference in the band intensity of β-turn among all samples (*p* > 0.05). However, a significantly lower intensity of antiparallel bands was found in 5–30, compared with the others (*p* < 0.05), suggesting lesser looseness in structure of the samples treated with the mildest SV conditions. Moreover, it was found that the ratio of amide III (1338 cm^−1^), representing collagen content, of samples treated at 60 °C was lower than samples treated at 50 °C, suggesting the higher temperature used for SV cooking can accelerate the degradation of connective tissue. This agreed well with the lower collagen content when the fish was cooked at 60 °C (Figure 1c), which helps to indicate its tenderness more.

### 3.3. Texture-Liking Score

The mean acceptability score for the texture attribute of SV-cooked tilapia processed by various conditions is shown in Table 1. Normally, for a 9-point hedonic scale, a mean liking score of 7.00 or higher is usually associated with a highly acceptable sensory quality [36]. The results demonstrated that the highest texture-liking score was obtained in 6–45, which was 7.59 (*p* < 0.05), suggesting the high acceptability of this product. However, there was no difference among other samples (*p* > 0.05). Based on the interesting physicochemical properties of various SV samples, it was found that SV-cooked tilapia meat with an increase in temperature from 50 to 60 °C and an increase in the time of cooking from 30 to 45 to 60 min led to an increased tenderness, as indicated by the lower shear force or hardness. However, juiciness was decreased with both the increase in temperature and time, as indicated by the lower WHC and higher cooking loss (Table 1). Thus, it may be concluded that SV tilapia meat cooked at 60 °C for 45 min had the most preferable meat texture, and these conditions can improve the meat’s tenderness to the expected consumer level. The significantly lower texture-liking score of 6–60, compared with 6–45, may explain that the prolonged processing time of up to 1 h resulted in too much tenderness over the expected level and may be associated with lower juiciness, to some extent.

### 3.4. Principal Component Analysis

**Figure 5:**
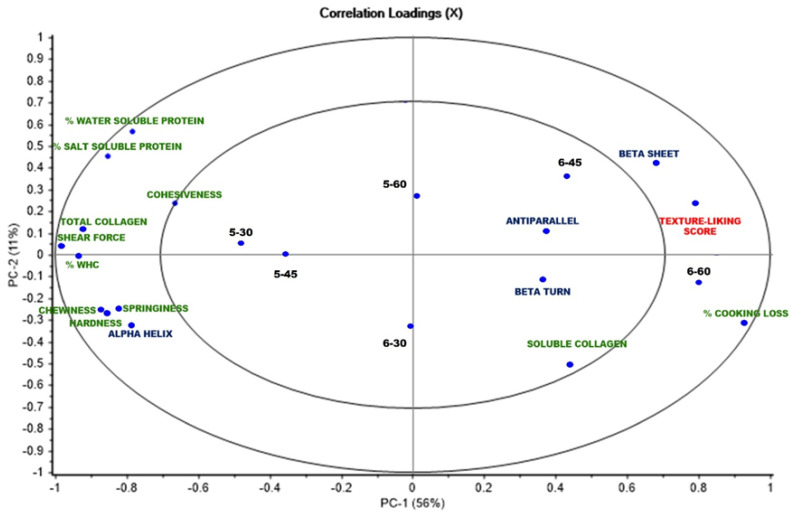
PCA score (samples, black color) and correlation loading (crucial physicochemical properties (green color), secondary protein structure (blue color), and texture-liking score (red color)) plots of tilapia fillet processed under various SV conditions.

The relationship between some crucial physicochemical properties, FTIR spectra, and texture-liking scores of all samples, for which the PC explained about 67% of the total variability of all data, is demonstrated as shown in Figure 5. It was noticed that the score plot of PC1 (vertical axis), which explained a variance of 56%, clearly discriminated against samples treated at 50 °C and 60 °C. From the left side along PC1, an association between the α-helix, the texture parameters from TPA, WHC, and the shear force, as well as water- and salt-soluble proteins was observed, which correlated with 5–30 and 5–45. This suggested that some physicochemical properties, including WHC, shear force, or texture characteristics, were governed by protein degradation, including sarcoplasmic proteins, myofibrils, or connective tissue occurring when heated up at 50 °C. However, these parameters were negatively correlated with texture-liking score, which appears on the right side along PC1. In contrast, it was found that samples including 6–45 and 6–60 had a positive correlation with the texture-liking score, which was also associated with the β-sheet, β-turn, or antiparallel structure of protein, insoluble collagen content, and cooking loss. This suggested that more severe SV conditions may lead to the partial aggregation of proteins and create some toughness, somehow, at the preferred level, thus resulting in the higher texture-liking score. A strong correlation between 6–60 and cooking loss was observed. Higher cooking loss may lead to a loss of juiciness in fish meat. This may describe the lower texture-liking score of this sample, compared with 6–45. Moreover, the PC2 (horizontal axis), which explained a lower variance percentage (11%), revealed that 6–45 had a higher correlation with the texture-liking score than 6–60. Therefore, the PCA results demonstrated that a temperature of 60 °C and a duration of 45 min seem to be the optimal conditions for SV-cooked tilapia in order to access the best quality of the product, particularly in the texture dimension.

## 4. Conclusions

Different SV temperatures (50–60 °C) and cooking times (30–60 min) applied to tilapia fillets can impact meat quality, particularly textural characteristics, differently. More severe SV conditions contributed to more protein degradation (of both myofibrils and connective tissue), resulting in the improvement of meat tenderness. However, a reduction in juiciness, as well as an increase in cooking loss, were among the negative effects when using a higher temperature or prolonged processing time in SV cooking. In this study, SV-cooked tilapia prepared at 60 °C for 45 min showed the best results for textural characteristics and acceptability, suggesting the optimal conditions for this product. These findings have important implications for the seafood industry and for consumers, since SV is becoming increasingly popular and is a convenient and reliable method to produce perfectly cooked, juicy tilapia in the home, restaurants, catering, and industry. However, shelf-life evaluation and pathogenic bacteria validation should be further studied to warrant/clarify the product quality and stability during storage.

## Figures and Tables

**Table 1:** Physicochemical properties and sensory scores of tilapia fillet processed under various SV conditions.

Parameters	5–30	5–45	5–60	6–30	6–45	6–60
WHC (%)	82.75 ± 1.53 ^a^	79.24 ± 0.94 ^b^	79.32 ± 1.38 ^b^	77.93 ± 2.08 ^b^	74.54 ± 1.62 ^c^	70.32 ± 2.42 ^d^
Cooking loss (%)	7.33 ± 0.25 ^d^	7.93 ± 0.19 ^c^	8.68 ± 0.26 ^b^	8.06 ± 0.17 ^c^	8.81 ± 0.20 ^b^	9.31 ± 0.19 ^a^
Shear force (N)	9.25 ± 0.23 ^a^	9.08 ± 0.14 ^a^	8.52 ± 0.18 ^b^	8.08 ± 0.30 ^c^	7.85 ± 0.28 ^c^	7.98 ± 0.26 ^c^
TPA						
Hardness (N)	618.96 ± 31.78 ^a^	632.81 ± 24.25 ^a^	526.96 ± 21.04 ^b^	444.64 ± 28.24 ^c^	437.43 ± 28.61 ^c^	372.79 ± 22.99 ^d^
Chewiness (N)	164.25 ± 6.72 ^b^	181.03 ± 4.59 ^a^	133.01 ± 5.97 ^c^	117.60 ± 6.68 ^d^	87.00 ± 3.98 ^e^	89.21 ± 1.51 ^e^
Springiness (mm)	0.71 ± 0.01 ^a^	0.70 ± 0.01 ^a^	0.71 ± 0.02 ^a^	0.67 ± 0.02 ^b^	0.62 ± 0.04 ^c^	0.66 ± 0.03 ^b^
Cohesiveness	0.38 ± 0.04	0.41 ± 0.07	0.36 ± 0.03	0.39 ± 0.04	0.33 ± 0.04	0.35 ± 0.05
Texture-liking score	6.76 ± 0.35 ^b^	6.70 ± 0.41 ^b^	6.72 ± 0.34 ^b^	6.94 ± 0.24 ^b^	7.59 ± 0.15 ^a^	7.11 ± 0.29 ^b^

Mean ± SD. Different lowercase superscripts in the same row indicate the significant difference (*p* < 0.05).

**Table 2:** Ratio of biomolecules determined by SR-FTIR and secondary protein structure after curve-fitting of tilapia fillet processed under various SV conditions.

Interested Spectra (Wavenumber)	% Integral Area					
	5–30	5–45	5–60	6–30	6–45	6–60
Biomolecules (% integral area)						
Lipids (3000–2800 cm^−1^)	13.00 ± 0.12 ^a^	12.68 ± 0.13 ^bc^	12.46 ± 0.15 ^c^	12.00 ± 0.02 ^d^	12.75 ± 0.25 ^ab^	12.75 ± 0.08 ^ab^
Ester lipids (1740 cm^−1^)	0.23 ± 0.02 ^b^	0.15 ± 0.00 ^d^	0.17 ± 0.01 ^c^	0.25 ± 0.01 ^a^	0.14 ± 0.01 ^d^	0.07 ± 0.00 ^e^
Amide I (1700–1600 cm^−1^)	48.50 ± 0.26 ^ab^	48.06 ± 0.77 ^b^	48.36 ± 0.49 ^ab^	49.07 ± 0.36 ^a^	48.36 ± 0.31 ^ab^	48.50 ± 0.56 ^ab^
Amide II (1600–1500 cm^−1^)	27.26 ± 0.10 ^b^	26.31 ± 0.53 ^c^	28.19 ± 0.44 ^a^	27.11 ± 0.18 ^b^	27.44 ± 0.41 ^b^	27.00 ± 0.33 ^b^
Amide III (1338 cm^−1^)	6.38 ± 0.14 ^a^	6.42 ± 0.26 ^a^	4.99 ± 0.20 ^c^	5.83 ± 0.19 ^b^	5.63 ± 0.80 ^b^	5.84 ± 0.24 ^b^
Carbohydrates (1250–900 cm^−1^)	4.62 ± 0.29 ^b^	5.66 ± 0.23 ^a^	5.83 ± 0.09 ^a^	5.73 ± 0.15 ^a^	5.67 ± 0.18 ^a^	5.84 ± 0.04 ^a^
Secondary protein structure (% curve fitting)						
α-helix (1640–1670 cm^−1^)	50.42 ± 1.79 ^a^	49.27 ± 1.26 ^a^	49.66 ± 0.50 ^a^	46.63 ± 1.06 ^b^	45.77 ± 1.94 ^b^	45.10 ± 1.43 ^b^
β-sheet (1620–1640 cm^−1^)	22.66 ± 1.06 ^b^	22.87 ± 1.21 ^b^	22.43 ± 0.40 ^b^	24.36 ± 1.03 ^a^	25.82 ± 1.36 ^a^	25.71 ± 1.69 ^a^
β-turn (1672, 1614 cm^−1^)	15.54 ± 0.41	15.76 ± 0.43	15.61 ± 0.40	16.02 ± 0.67	15.68 ± 0.76	16.85 ± 1.57
Antiparallel (1680–1695 cm^−1^)	11.38 ± 0.26 ^b^	12.10 ± 0.31 ^a^	12.30 ± 0.45 ^a^	12.99 ± 0.38 ^a^	12.73 ± 0.45 ^a^	12.35 ± 0.48 ^a^

Mean ± SD. Different lowercase superscript letters in the same row indicate the significant difference (*p* < 0.05).

## Data Availability

The data presented in this study are available on request from the corresponding author (J.P).

## Supplementary Materials

All supplementary data (Figure S1: Temperature profiles of heating medium and core fillets at 50 ℃ (a) and 60 ℃ (b) for various times during SV-cooking; Table S1: Physicochemical properties of raw tilapia (before applied with SV-cooking) can be downloaded at: https://www.mdpi.com/article/10.3390/foods12030568/s1.
