# Supplementary material for: Investigating the Effect of Various Sous-Vide Cooking Conditions on Protein Structure and Texture Characteristics of Tilapia Fillet Using Synchrotron Radiation-Based FTIR
Source: Foods. 2023 Jan 28;12(3):568. doi: 10.3390/foods12030568 (PMC9914579; doi:10.3390/foods12030568)
Supplement: Supplementary file 1 [file foods-12-00568-s001.zip › foods-2156937-supplementary.pdf]

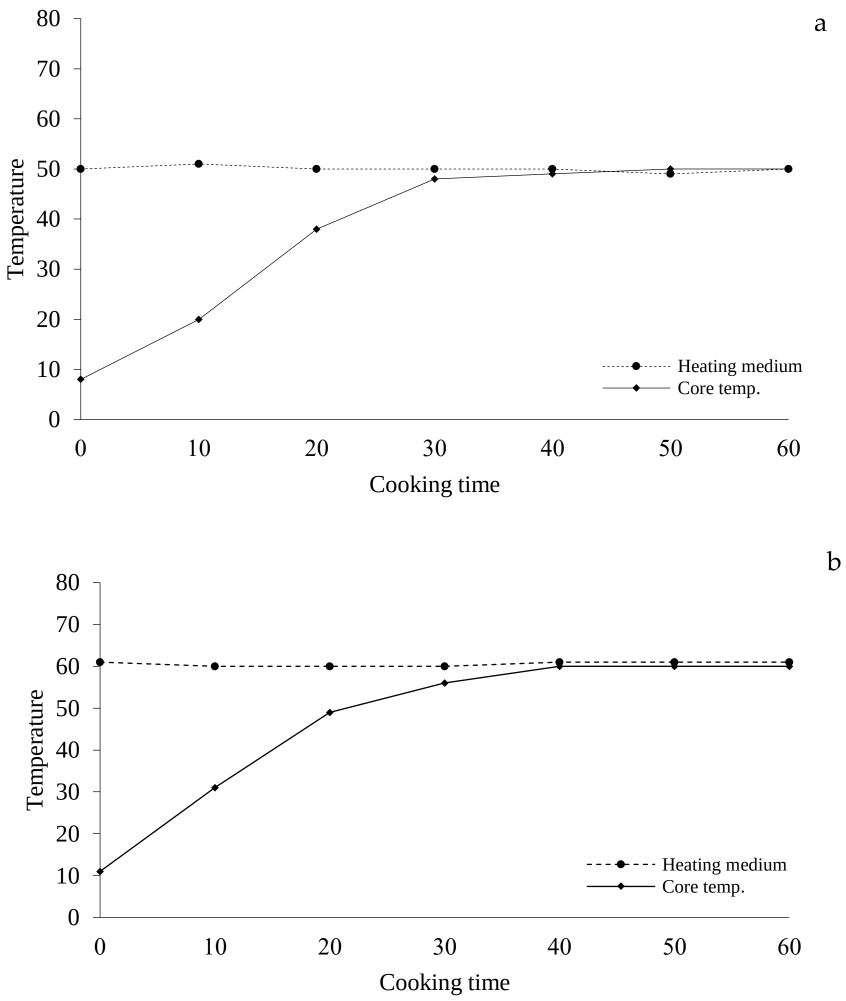

Figure S1. Temperature profiles of heating medium and core fillets at 50°C (a) and 60°C (b) for various times during SV-cooking.

Table S1. Physicochemical properties of raw tilapia (before applied with SV-cooking).

Parameters	Raw tilapia
WHC (%)	93.63±2.55
Shear force (N)	15.55±0.70
TPA	
Hardness (N)	1,368.01±103.25
Chewiness (N)	666.29±30.15
Gumminess (N)	713.32±26.23
Springiness (mm)	0.70±0.02
Cohesiveness	0.40±0.05
Water-soluble protein (%)	5.23±0.45
Salt-soluble protein (%)	10.06±2.01
Total collagen (mg/g)	12.19±0.92
Insoluble collagen (mg/g)	3.19±0.29

Mean ± SD. Different lowercase superscripts in the same row indicate the significant difference ($P<0.05$).
